# Experimental Study on the Microfabrication and Mechanical Properties of Freeze–Thaw Fractured Sandstone under Cyclic Loading and Unloading Effects

**DOI:** 10.3390/ma17102451

**Published:** 2024-05-19

**Authors:** Taoying Liu, Wenbin Cai, Yeshan Sheng, Jun Huang

**Affiliations:** 1 School of Resource and Safety Engineering, Central South University, Changsha 410083, China; 2 Guangxi Liubin Expressway Construction and Development Co., Ltd., Nanning 530023, China

**Keywords:** freeze–thaw cycles, rock mechanics, cyclic loading and unloading, microstructure

## Abstract

A series of freeze–thaw cycling tests, as well as cyclic loading and unloading tests, have been conducted on nodular sandstones to investigate the effect of fatigue loading and freeze–thaw cycling on the damage evolution of fractured sandstones based on damage mechanics theory, the microstructure and sandstone pore fractal theory. The results show that the number of freeze–thaw cycles, the cyclic loading level, the pore distribution and the complex program are important factors affecting the damage evolution of rocks. As the number of freeze–thaw cycles rises, the peak strength, modulus of elasticity, modulus of deformation and damping ratio of the sandstone all declined. Additionally, the modulus of elasticity and deformation increase nonlinearly as the cyclic load level rises. With the rate of increase decreasing, while the dissipation energy due to hysteresis increases gradually and at an increasing rate, and the damping ratio as a whole shows a gradual decrease, with a tendency to increase at a later stage. The NRM (Nuclear Magnetic Resonance) demonstrated that the total porosity and micro-pores of the sandstone increased linearly with the number of freeze–thaw cycles and that the micro-porosity was more sensitive to freeze–thaw, gradually shifting towards meso-pores and macro-pores; simultaneously, the SEM (Scanning Electron Microscope) indicated that the more freeze–thaw cycles there are, the more micro-fractures and holes grow and penetrate each other and the more loose the structure is, with an overall nest-like appearance. To explore the mechanical behavior and mechanism of cracked rock in high-altitude and alpine areas, a damage model under the coupling of freeze–thaw-fatigue loading was established based on the loading and unloading response ratio theory and strain equivalence principle.

## 1. Introduction

It is estimated that nearly 50% of the world’s seasonally frozen and permafrost areas are located around the globe [1]. With the rapid development of construction projects in cold regions, rock damage caused by freezing and thawing affects the durability and longevity of geostructures, especially those constructed in areas frequently affected by climate change [2,3]. Furthermore, excessively low temperatures for engineering building cause significant freeze–thaw disaster difficulties; the future development of a large number of cold locations will encounter ultra-low temperature frozen rock adverse geological environments [4]. Under the action of a freeze–thaw cycle, the permeability of the fractured rock mass is reduced, and water penetrates into it. After freezing, water ice phase change and volume expansion make the volume of water expand by about 9%, resulting in frost heave pressure and aggravating the development, initiation, convergence, expansion and penetration of rock mass cracks [5,6], which are often used as a cyclic or fatigue load, and their mechanical properties are very different from those under static load, such as tunnel blasting excavation and support processes, deep oil storage tank liquid circulation repeated input and output and the excavation and support of high slopes. The mining face is carried out alternately, which involves cyclic and repeated fatigue stresses. The deformation strength characteristics and fracture damage characteristics of rock are closely related to the stress path and loading history. Meanwhile, the macro and micro crack development and the expansion evolution of fractured rock mass are greatly affected by periodic loads.

At present, with the continuous development of science and technology, numerous scholars have observed the microscopic damage of rocks after freeze–thaw cycles with the help of advanced equipment, such as NMR, scanning electron microscopes, CT, rock wave velocity meters, morphological scanners, etc. Niu Caoyan et al. [7] investigated the effect of freeze–thaw cycling on the porosity and fracture surface microstructure of rocks with the aid of nuclear magnetic resonance (NMR) and scanning electron microscopy (SEM), showing that the higher the number of freeze–thaw cycles, the lower the porosity of the rock. Li Jielin [8,9,10] classified the internal pores of rocks into small, medium and large pores according to the pore size distribution characteristics, and it was obvious that small and medium pores kept developing and large pores kept expanding under the effect of freezing and swelling pressure, and it was also found that the NMR porosity and T2 spectral area of rocks showed exponentially decreasing distribution characteristics with the uniaxial compressive strength of rocks. X-ray CT scans are mostly utilized in medicine and industries. It is a method of inspecting the interior deterioration of materials. Benjamin K. Blykers scanned mineral building materials using X-ray dark-field imaging (DFI) to analyze the micropores inside the materials through dark-field signals [11]. CT technology is increasingly being utilized to examine the interior damage features of rocks. Koorosh Abdolghanizadeh et al. [12], Wang Y et al. [13] and Liu Hui et al. [14] compared the damage characteristics of rocks under different freeze–thaw cycles by the CT technique, and the study showed that with the higher number of freeze–thaw cycles, the CT value increased, the internal crack development of rocks kept expanding, the structure was obviously defective and deteriorated and the internal damage was boosted.

The deep rock is not only in the state of high ground stress but is also affected by stress disturbances such as mining, drilling and blasting, which cause stress redistribution, internal structure changes and even engineering instability. There are various types of cyclic loading and unloading, including equal amplitude cyclic loading and unloading, graded cyclic loading and unloading, variable lower limit cyclic loading and unloading and constant lower limit cyclic loading and unloading. Liu Xiangyu et al. [15] carried out equal-amplitude cyclic and step-by-step primary cyclic unloading tests on siltstone to compare the pore expansion, crack production and crack evolution patterns in the damage phase under the two unloading methods. The test results show that the porosity of the two types of unloaded rocks tends to increase first and then decrease, and the rock samples are mainly damaged by shear; the damage to the equal-amplitude cyclic unloading rock samples occurs during the loading stage, displaying instantaneous damage characteristics, whereas the damage to the rock samples arises during the unloading stage, displaying small amplitude oscillation damage features. Wang Tianzuo et al. [16] applied acoustic emission to compare the mechanical properties of constant lower limit cyclic loading and unloading and variable lower limit cyclic loading and unloading under uniaxial compression tests and found that constant lower limit cyclic loading and unloading increased the rock strength by 6.5%. The complexity of fatigue damage is so significant that the evolution of the damage is difficult to describe in terms of general mechanical theory. As the deformation and damage of rocks are essentially caused by energy release, energy analysis is an important and critical method for revealing the evolution of damage in rocks under disturbed stress conditions. To determine the total rock energy, elastic strain energy and dissipation energy at various fracture dips, Peng Kang et al. [17] performed stress gradient continuous cycle loading and unloading studies on sandstones with varied fracture dips. Ju Wang et al. [18,19] investigated the characteristics of energy storage and dissipation, damage evolution and failure modes of red sandstone under discontinuous multistage fatigue (DMLF) loading at different fracture dips, and the results showed that the total energy, elastic energy and dissipation energy showed a quadratic polynomial function increase with increasing stress.

In summary, NMR, SEM and CT can effectively observe the microscopic damage of rocks after freeze–thaw cycles. In the meantime, there is a lack of research on the damage evolution of fractured rock under freeze–thaw-fatigue unloading loading coupling effects. Consequently, based on the results of previous studies [20,21], NMR and SEM are used to investigate rock damage under coupled freeze–thaw-fatigue loading as a means of enhancing the rigor of the results to complement the gaps in previous studies. It is not only an important research frontier in the discipline but also a significant social and engineering background and is of great practical significance in predicting the occurrence of geoengineering hazards in alpine and high-altitude areas affected by disturbed loading.

## 2. Experimental System and Methods

**Figure 1:**
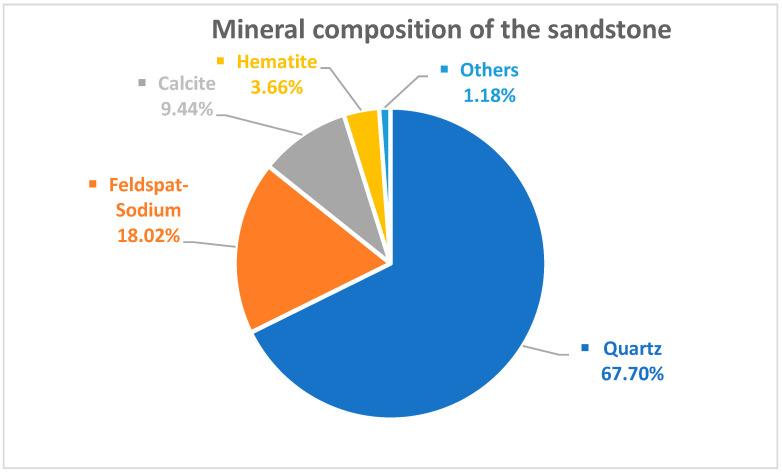
Mineral composition of the sandstone.

**Figure 2:**
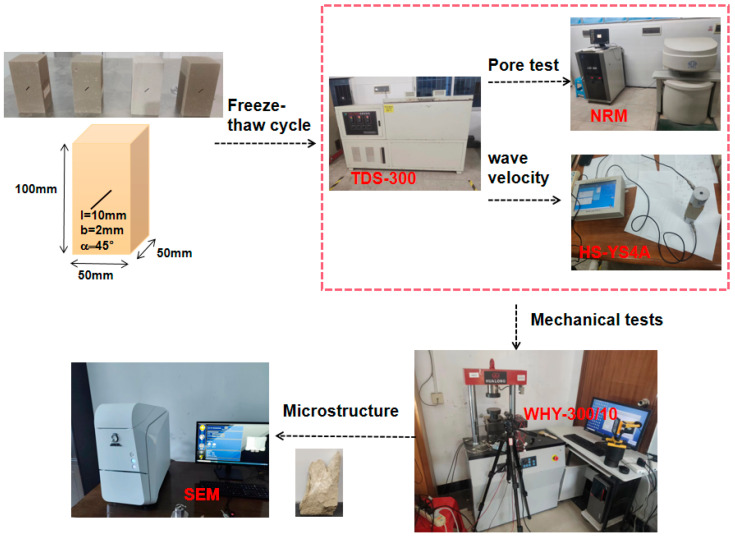
Test systems.

This experiment was designed according to the Code for rock tests in water and hydropower projects (SL/T 264-2020). A yellow sandstone from a mine was selected and machined into a 50 mm × 50 mm × 100 mm square specimen with a 10 mm long, 2 mm wide, 45° inclined prefabricated fracture through the middle. In this experiment, three sandstone specimens were tested at each set of freeze–thaw cycles, and the statistical data obtained from the experiment were averaged to minimize experimental errors [22]. The basic mechanical parameters of sandstone are shown in Table 1, and the mineralogical composition of the sandstone is shown in Figure 1. The sandstone was saturated with water before the test began and then placed in a TDS-300 freeze–thaw tester to start the freeze–thaw damage test. An AiniMR-150 NMR (Shanghai Newmy Electronics Technology Co., Ltd., Shanghai, China) instrument was used to measure the porosity changes before and after the freeze–thaw cycle and an HS-YS4A rock wave velocity meter (Beijing Haifuda Technology Co., Ltd., Beijing, China) was used to determine the wave velocity of sandstone before and after freeze–thaw damage, followed by a WHY-300/10 microcomputer-controlled electro-hydraulic servo universal testing machine (Shanghai Hualong Test Instruments Co., Ltd., Shanghai, China) carrying out the cycle plus unloading test and then removing the damaged fragments for SEM microstructure observation. The test process is shown in Figure 2.

**Figure 3:**
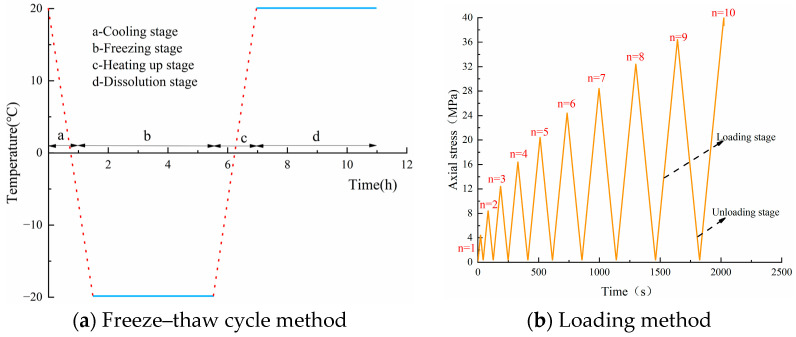
Test method.

The freeze–thaw cycle was executed in five sets of 0, 20, 40, 60 and 80 freeze–thaw cycles, with three specimens made under each test condition. For each cycle, the freezing time is 240 min, the melting time is 240 min and the freezing and melting temperatures are −20 °C and 20 °C, respectively. It takes about 1.5 h for each cycle to decrease from a normal temperature of 20 °C to −20 °C and then from a freezing temperature to melting. The cumulative duration of a freeze–thaw cycle is about 11 h, as shown in Figure 3a. The fatigue test was performed in a constant down-line cyclic loading and unloading mode with force control and a loading rate of 0.5 kN/s; the loading mode was 0 kN→11 kN→1 kN→21 kN→1 kN→31 kN→1 kN…, as indicated in Figure 3b.

## 3. Analysis of Experimental Results

### 3.1. Stress–Strain Curves

**Figure 4:**
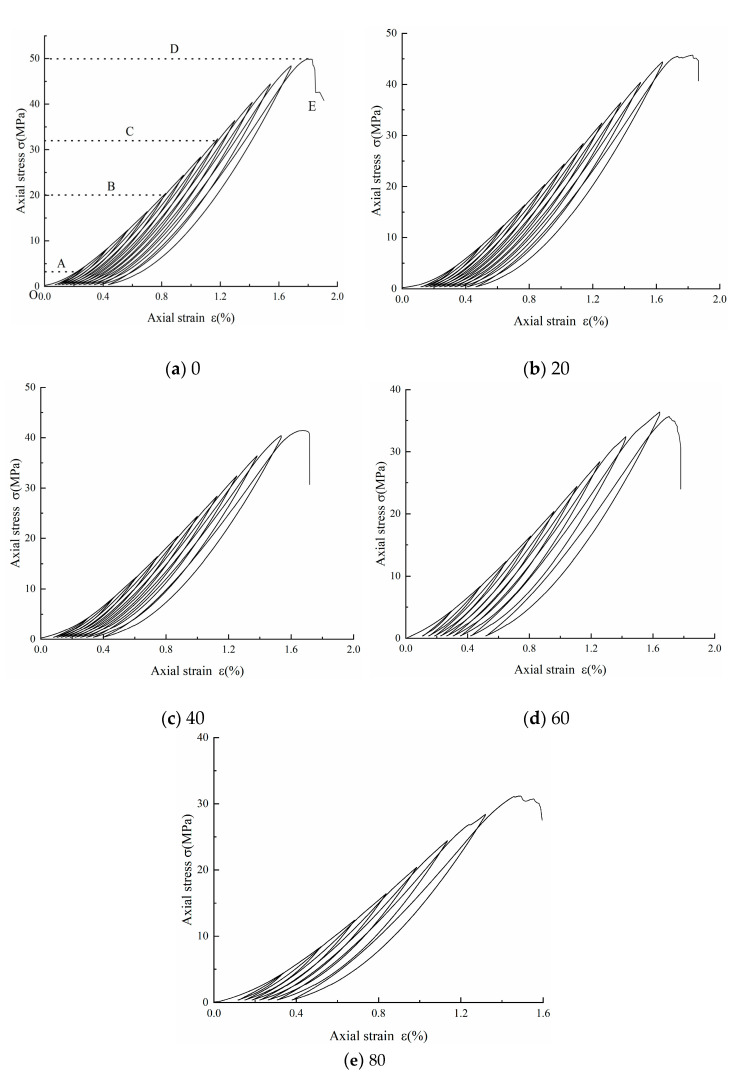
Stress–strain curve.

Figure 4 shows the cyclic stress–strain curves for the fractured sandstone at 0, 20, 40, 60 and 80 freeze–thaw cycles. The hysteresis curve spacing of the specimens shows an overall “dense and sparse” pattern, and the higher the number of freeze–thaw cycles, the sparser the overall hysteresis curve spacing. In the early stages of loading, the peak value of each stage of loading was almost on the same curve while gradually shifting in the direction of increasing strain, and this curve was found by most scholars to coincide with the uniaxial compression curve of the specimen, while in the late stages of loading, the curve deviated significantly from the historical stress–strain curve [23,24], which indicates that excessive fatigue loading in the early stages led to the accumulation of damage inside the specimen and the gradual weakening of the bearing capacity.

The evolution of the stress–strain curve throughout the cyclic loading and unloading test of the post-freeze–thaw fractured rock consists of five stages, as shown in Figure 4a. There are five stages in the evolution of the stress–strain curve during the cyclic loading and unloading test of the post-freeze–thaw fractured rock [25].

(1) The OA compacting stage: This stage is the initial loading stage of the specimen, with the increase in axial stress, the specimen internal microcracks and pore compacting. The curve shows non-linear growth, and it was also found that with the increase in the number of freeze–thaw cycles, the faster the change in axial stress, indicating that there is freeze–thaw action due to the water in the rock micro-porosity. The water–ice phase change produces about 9% volume expansion, making the micro-porosity further increase.
(2) The AB elastic deformation stage: This stage keeps the curve approximately straight up as the axial force continues to increase. This stage is elastic deformation and can be recovered.
(3) The BC crack stable expansion stage: As the axial force increases, the curve exhibits a non-linear growth trend and the microcracks within the specimen begin to expand with a gradual increase in density and in the direction of the maximum principal stress.
(4) The CD crack instability expansion stage: In this stage, with the further increase in the axial force, cracks occur and gradually gather into nuclei, the micro-crack expansion rate increases rapidly and eventually reaches the peak strength and the specimen is damaged.
(5) The DE damage stage: The deformation of the sandstone increases rapidly under the continuous action of axial stress, and after reaching the compressive strength of the specimen, the load-bearing capacity decreases rapidly and the stress–strain curve falls off rapidly. There is obvious brittle damage of the specimen, which maintains a certain residual strength due to the presence of shear strength and friction on the fracture surface of the specimen.

### 3.2. Mechanical Parameters

The mass and wave velocity of sandstone before and after freezing and thawing were measured, and the damage of the mass and wave velocity was calculated, as shown in Equations (1) and (2), where $D_{m}$ is the mass damage factor, $\bar{m_{0}}$ and $\bar{m_{i}}$ are the average mass (kg) of the sandstone before and after the freeze–thaw cycle, respectively, $D_{v}$ is the wave velocity damage factors and $V_{i}$ is the mean wave velocities (m/s) of the sandstone before and after the freeze–thaw cycle, respectively.

(1) $$D_{m}=\frac{\bar{m_{0}}-\bar{m_{i}}}{\bar{m_{0}}}$$

(2) $$D_{v}=\frac{\bar{V_{0}}-\bar{V_{i}}}{\bar{V_{0}}}$$

**Figure 5:**
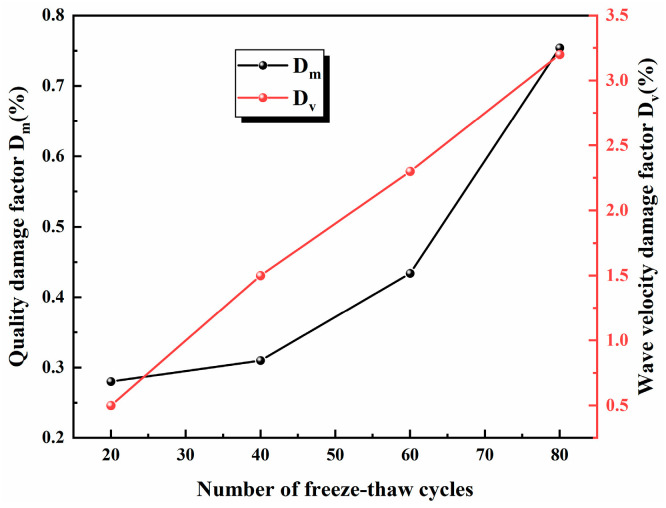
Damage factor.

Figure 5 represents the relationship between the mass and wave damage factor after freeze–thaw cycles in sandstone and the number of freeze–thaw cycles. As the number of freeze–thaw cycles increases, the overall mass and wave velocity damage factors show a pattern of increasing. The low number of freeze–thaw cycles has a small effect on sandstone mass loss, while the high number of freeze–thaw cycles leads to a sudden increase in the mass damage factor and more mass loss, indicating that dislocation and movement between mineral grains within the sandstone under the action of water–ice phase change can lead to particle loss and rock chips falling off the surface. The wave speed is a reflection of the extent of internal defects (microcracks, pores and joints, etc.) in the rock; the lower the wave speed, the more serious the internal defects [26]. It is not difficult to find that with the increase in the number of freeze–thaw cycles, the wave velocity damage factor rises almost linearly, and the internal deterioration degree of sandstone is deepened. Moreover, it is found in the test that cracks appear on the prefabricated crack surface of sandstone after freeze–thaw damage, as well as the phenomenon of a frozen crisp at the edge.

**Figure 6:**
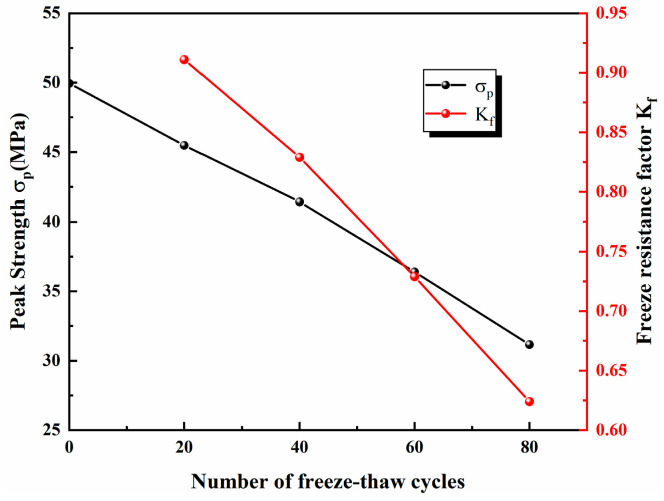
Relationship between the peak intensity and the number of freeze–thaw cycles.

The damage strength and damage rating of each sandstone are shown in Table 2. The sandstone with no freeze–thaw damage reaches a load cycle rating of 12, and as the number of freeze–thaw cycles gradually increases, the damage rating gradually decreases, culminating in a sandstone damage rating of 7 after 80 freeze–thaw cycles, a reduction of 5 grades compared to that with no freeze–thaw. As can be seen in Figure 6, the peak strength of the sandstone decreased almost linearly from 49.95 MPa to 31.17 MPa, a decrease of 37.6%, indicating that freeze–thawing has a significant effect on the deterioration of the sandstone bearing capacity. To further evaluate the degree of strength loss before and after freeze–thaw cycles, the freeze–thaw coefficient is often used. The larger the value, the less freeze–thaw damage and the better the resistance to freezing, and vice versa. As the number of freeze–thaw cycles increases, the freezing resistance of the sandstone decreases linearly from 0.911 to 0.624, a decrease of 31.5%, indicating that repeated freeze–thawing exacerbates the evolution of the sandstone damage and weakens the internal cementation between the grains.

(3) $$K_{f}=\frac{\bar{R_{f}}}{\bar{R_{s}}}$$

where $K_{f}$ denotes the frost resistance factor and $\bar{R_{f}}$ and $\bar{R_{s}}$ denote the peak strength after freeze–thaw cycles and the peak strength before freeze–thaw cycles, respectively.

(4) $$\varepsilon_{s}=\varepsilon_{e}+\varepsilon_{cr}$$

where $\varepsilon_{s}$ is the total strain in each phase, $\varepsilon_{e}$ is the elastic strain in each phase and $\varepsilon_{cr}$ is the plastic strain in each stage.

(5) $$E_{e}=\frac{\sigma_{max}-\sigma_{min}}{\varepsilon_{e}}$$

(6) $$E_{d}=\frac{\sigma_{max}-\sigma_{min}}{\varepsilon_{s}}$$

where $E_{e}$ is the modulus of elasticity for each phase, $E_{d}$ is the modulus of deformation in each stage and $\sigma_{max}$ and $\sigma_{min}$ are the maximum and minimum stresses in each stage, respectively.

(7) $$\lambda =\frac{A_{R}}{4\pi A_{s}}$$

**Figure 7:**
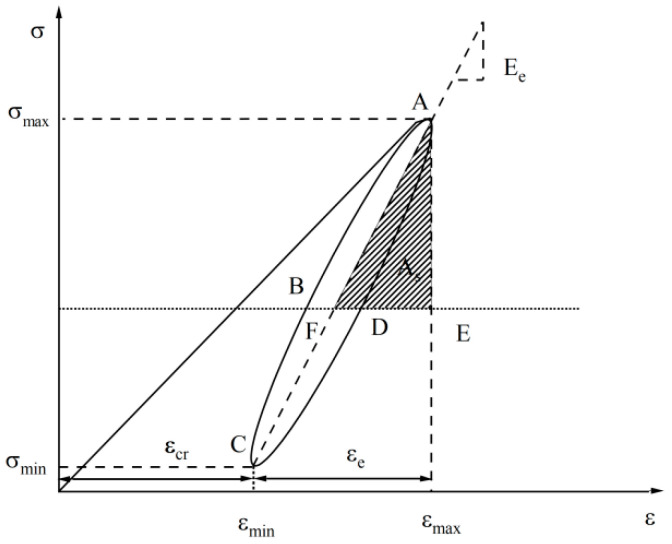
Schematic diagram of the hysteresis loop of the stress–strain curve.

Figure 7 represents the stress–strain hysteresis curve and calculates the modulus of elasticity, modulus of deformation and damping ratio, respectively. λ is the damping ratio, $A_{R}$ is the area of hysteresis circle ABCD (kJ/m^3^) and $A_{s}$ is the area of the triangle AEF (kJ/m^3^).

**Figure 8:**
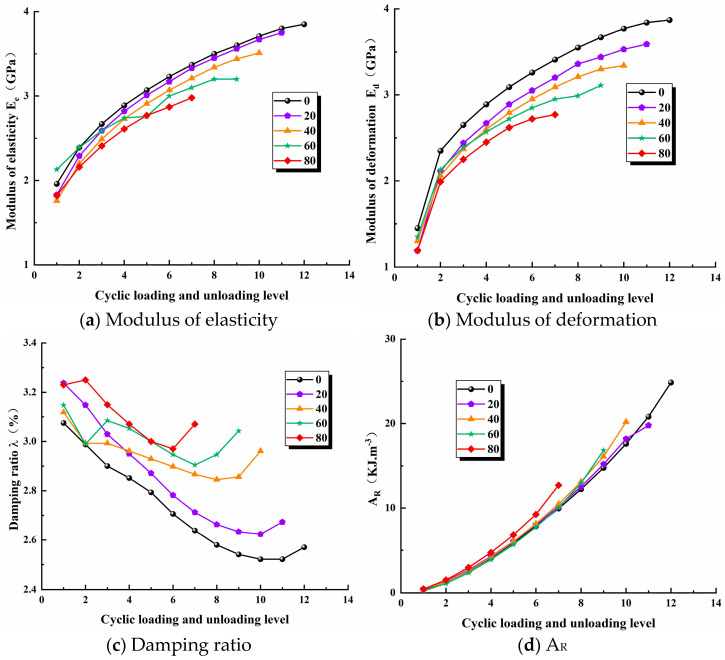
Mechanical parameters.

Figure 8 represents the modulus of elasticity, modulus of deformation, damping ratio and area of hysteresis circle ABCD as a function of the number of freeze–thaw cycles. The modulus of elasticity reflects the ability of the sandstone to deform elastically, while the modulus of deformation reflects the deformation energy of the sandstone as a whole, including both elastic and plastic deformation [27,28]. It can be seen in Figure 8a,b that the modulus of elasticity and modulus of deformation gradually increase with increasing cyclic load levels at a constant number of freeze–thaw cycles, but the rate of increase is gradually slow, indicating that the load size increases step by step and the overall deformation increases. While the deformation at each level decreases, the load-bearing capacity of the sandstone is continuously weakened, internal microcracks expand and penetrate and damage gradually accumulates; at the same time, the deformation and resilience of the sandstone is continuously weakened and gradually turns into plastic damage. Additionally, the modulus of deformation increases abruptly when the cyclic load rating is increased from level 1 to level 2, mainly due to the compacting of the precast fractures and internal micro-cracks under the load. The higher the number of freeze–thaw cycles, the lower the modulus of elasticity and modulus of deformation of the sandstone. The main reason for this is that water enters the micropores of the sandstone, the water–ice phase change generates freezing pressure, followed by volume expansion, and repeated icing and melting increase the micro-pores, deteriorating the load-bearing capacity of the sandstone and leading to a significant reduction in the deformation capacity of the sandstone. It is also easy to see that sandstones with a high number of freeze–thaw cycles have a lower modulus of elasticity and modulus of deformation per load cycle class than sandstones with a low number of freeze–thaw cycles. This is because the greater the freeze–thaw damage to the sandstone under the same sized loading, the more the deformation and load-bearing capacity are weakened, resulting in the decay of the modulus of elasticity and modulus of deformation per level.

The damping ratio is a significant reflection of the mechanical properties of the rock under the cyclic loading of regional earthquakes. Under cyclic loading, the loading and unloading curves of sandstone do not coincide and exhibit hysteresis curves, i.e., reflecting the axial in damped vibration, while the area and shape of the hysteresis circle reflect the magnitude of dissipation energy generated by internal damage in the rock during a load cycle [29,30]. In Figure 8c, it can be seen that as the load cycle level increases, the damping ratio as a whole shows a pattern of gradually decreasing while having an increasing trend at a later stage. The author believes that as the load cycle level increases, the internal damage to the sandstone gradually accumulates, but the rate of damage development is slower, and when the sandstone damage load is reached, a large number of internal cracks in the sandstone expand, aggregate and penetrate, surface macro cracks are produced, and plastic deformation is more likely to occur, thus leading to a tendency for the damping ratio to increase at a later stage [31]. The higher the number of freeze–thaw cycles, the lower the damping ratio, and it fluctuates between 2.52% and 3.25%. This is mainly due to the fact that freeze–thaw aggravates the damage inside the sandstone and increases the porosity inside, resulting in greater plastic deformation of the sandstone and, hence, a greater damping ratio. As can be seen in Figure 8d, the dissipation energy due to hysteresis increases gradually and at an increasing rate as the cyclic load level increases. The difference in the magnitude of the dissipation energy release is initially small for sandstones subjected to different numbers of freeze–thaw cycles but gradually becomes larger in the later stages and increases abruptly under the final cyclic load. Similarly, more dissipative energy is released from sandstones subjected to a higher number of freeze–thaw cycles than from sandstones subjected to a lower number of freeze–thaw cycles at each load level. This is mainly due to the fact that the initial load is small, the number of internal microcracks is small and, therefore, the degree of damage is low, and the difference in the dissipation energy release is small for sandstones with different numbers of freeze–thaw cycles. This is when the dissipation energy increases dramatically. It is easy to explain that the higher the number of freeze–thaw cycles, the more porosity and micro-cracks are created in the sandstone and the higher the dissipation energy release per stage under the same load, while the sandstone undergoes fewer cycles and eventually the total dissipation energy release decreases due to the severe damage inside the sandstone exacerbated by freeze–thaw. The dissipative energy released from the sandstone for 0, 20, 40, 60 and 80 freeze–thaw cycles is 24.84 kJ/m^3^, 19.78 kJ/m^3^, 20.20 kJ/m^3^, 16.84 kJ/m^3^ and 12.70 kJ/m^3^, respectively. The increase in the number of freeze–thaw cycles from 0 to 80 reduces the dissipative energy release by 12.14 kJ/m^3^, a reduction of 48.87%; consequently, the freeze–thaw damage to sandstone is not negligible.

### 3.3. Microstructure

#### 3.3.1. Nuclear Magnetic Resonance

To better reflect the microscopic pore changes in freeze–thaw sandstone, different numbers of freeze–thaw cycles were plotted based on $T_{2}$ spectra. The transverse relaxation rate of NMR (Nuclear Magnetic Resonance) can be expressed by the following equation according to NMR theory.

(8) $$\frac{1}{T_{2}}=\rho_{2}\frac{s}{v}=\frac{\rho_{2}F_{2}}{r_{c}}$$

where $T_{2}$ is the lateral relaxation time (ms), $\rho_{2}$ is the surface relaxation strength, s is the pore surface area, v is the pore volume, $F_{2}$ is the core pore shape factor, usually a constant and pore shape dependent, and $r_{c}$ is the pore radius of the rock. Let $\rho_{2}F_{2}=C$ and take C = 10; then, Equation (8) becomes Equation (9) [31].

(9) $$r_{c}=CT_{2}=10T_{2}$$

**Figure 9:**
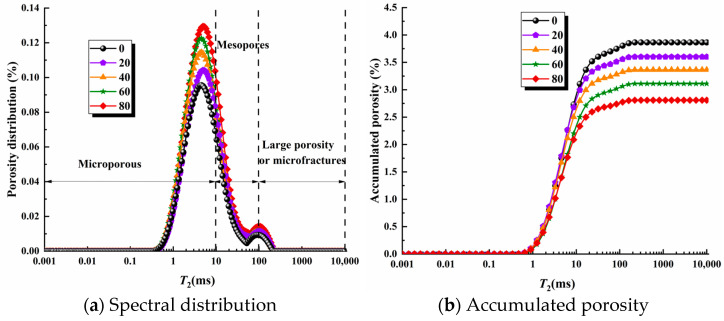
$T_{2}$ Spectral distribution and accumulated porosity.

According to the sandstone $T_{2}$, the spectral peak curves are divided into different pore types according to different pore distributions, and the author, based on the experimental capillary pressure measurement porosity radius grading method and combined with relevant literature, divided the red sandstone pore size into three intervals, i.e., small pores (r≤1 μm), medium pores (1 μm≤r≤10 μm) and large pores (r ≤10 μm) [10,32,33]. Combined with Equation (9), it can be seen that the transverse relaxation time $T_{2}$ spectrum is distributed in the range of 0–10 ms for small pores, 10–100 ms for medium pores and above 100 ms for large pores or micro-fractures. The spectra showed a bimodal pattern, as shown in Figure 9a. As can be seen in Figure 9b, the cumulative porosity is almost zero within the relaxation time of 1 ms, while the cumulative porosity increases dramatically when the relaxation time increases from 1 ms to 10 ms, after which the cumulative porosity rises slowly and finally reaches the equilibrium value. It can be found that the cumulative porosity starts to differ slowly after the time of 10 ms for different numbers of freeze–thaw cycles, and the higher the number of freeze–thaw cycles, the lower the cumulative porosity, which indicates that the deterioration damage of the pore structure by freeze–thaw is gradually enhanced.

**Figure 10:**
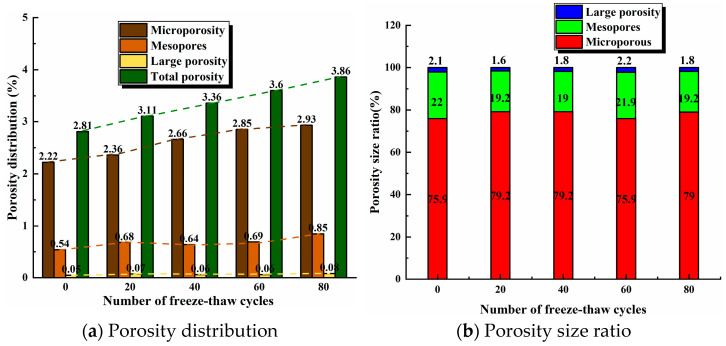
Changes in pore distribution with freeze–thaw cycles.

According to the above grading criteria, the different pore fractions of fracture dip 45° sandstone are counted and shown in Figure 10a. The total porosity and micro-porosity of the sandstone increased almost linearly as the number of freeze–thaw cycles increased from 0 to 80, the total porosity increased from 2.81% to 3.86%, an increase of 37.4%, and the microporosity increased from 2.22% to 2.93%, an increase of 32%, while the medium porosity increased to a lesser extent and the large porosity remained almost unchanged. The increase in mesoporosity was smaller, while the macroporosity remained almost unchanged and was less affected by freeze–thaw. The sandstone is dominated by micropores, which account for more than 70% of the total pores; see Figure 10b. Micro-pores develop rapidly under the influence of freeze–thaw, with the greatest increase in number, and are more sensitive to freeze–thaw, and the proportion of mesopores and macropores increases after 80 freeze–thaw cycles, indicating that the higher the number of freeze–thaw cycles, the sandstone gradually develops micropores into macropores, and the internal fine structure of the sandstone is gradually damaged.

Since the porosity distribution is irregular and complex, the use of a simple geometric formulation to describe it is a complete failure to provide insight into porosity. However, it can be studied using fractal theory, i.e., it can be characterized using a fixed non-integer dimension between Euclidean dimensions [34]. The fractal dimension is a quantitative parameter that describes the degree of irregularity of a fractal object. The degree of irregularity of a fractal object, and thus the complexity and irregularity of the pore structure, can be indirectly reflected by this parameter.

According to the fractal theory [35], the number n of pores with diameters larger than r satisfies the following functional relationship.

(10) $$n\left(>r\right)=\int_{r}^{r_{max}}I\left(r\right)dr=ar^{-D}$$

The volume of a pore with a pore size less than r is denoted as

(11) $$V\left(<r\right)=\int_{r_{min}}^{r}I\left(r\right)ar^{3}dr$$

where $r_{min}$ and $r_{max}$ are the minimum and maximum porosity, respectively, $I\left(r\right)$ is the pore size distribution density, a is a constant and D is the pore fractal dimension.

Combining Equations (10) and (11) yields

(12) $$V\left(<r\right)=\beta \left(r^{3-D}-r_{min}^{3-D}\right)$$

where β is a constant.

The cumulative pore volume fraction for pore sizes smaller than r is expressed as

(13) $$S_{V}=\frac{V\left(<r\right)}{V_{S}}=\frac{r^{3-D}-r_{min}^{3-D}}{r_{max}^{3-D}-r^{3-D}}$$

where $V_{S}$ is the total porosity.

Due to $r_{min}\ll r_{max}$, Equation (13) can be simplified as

(14) $$S_{V}=\frac{r^{3-D}}{r_{max}^{3-D}}$$

According to Equation (9), $T_{2}$ is proportional to r. Consequently, Equation (14) can be written as

(15) $$S_{V}={\left(\frac{T_{2}}{T_{2,max}}\right)}^{3-D}$$

where $T_{2,max}$ is the maximum relaxation time.

Both sides of Equation (15) are taken logarithmically.

(16) $$lgS_{V}=\left(3-D\right)lgT_{2}+\left(D-3\right)lgT_{2,max}$$

Therefore, the porosity fractal dimension can be obtained by taking the logarithm of the NMR technique porosity distribution curve and fitting a linear regression to Equation (16) with the slope of the regression curve as $\left(3-D\right)$.

**Figure 11:**
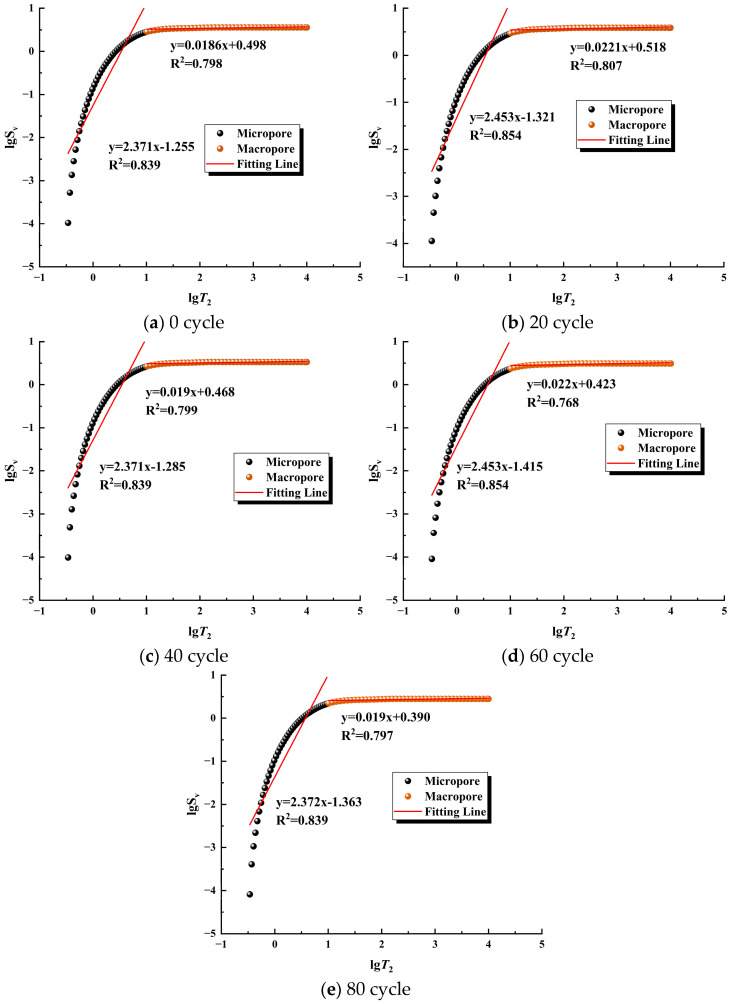
NMR fractal characteristics of the samples.

**Figure 12:**
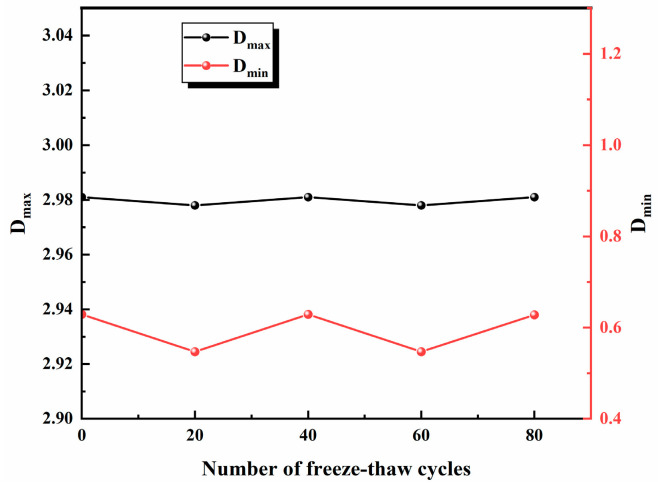
Changes in *D_max_* and *D_min_* under the freeze–thaw cycle.

Figure 11 represents the $lgS_{V}$ and $lgT_{2}$ relationship curve, and it can be found that the curve is not a straight line, indicating that there are obvious fractal differences between micropores and macropores. A linear fit was performed for the two parts with different numbers of freeze–thaw cycles, and it was found that the linear regression fit was better. $D_{min}$ and $D_{max}$ were used to represent the fractal dimensions of micropores and macropores, respectively, and the results are shown in Table 3. As can be seen in Figure 12, with the increase in the number of freeze–thaw cycles, $D_{max}$ almost did not change excessively, while $D_{min}$ produced a certain float. This indicates that the expansion under the action of a freeze–thaw force has a greater effect on micropores compared with macropores, so the study of the evolution of the freeze–thaw deterioration of sandstone needs to consider the state of micropore distribution and micropore complexity.

Because the size of sandstone pores, compared with other hard rock pore sizes, is larger, the most obvious effect of freeze–thaw on sandstone is the change in porosity. The freeze–thaw factor in Equation (17) can be used to describe the sandstone pore destruction process.

(17) $$W_{t}=\frac{1-P_{N}}{1-P_{0}}$$

where $W_{t}\;$ is the freeze–thaw damage factor. Only the freeze–thaw damage factor can not describe the sandstone freeze–thaw damage porosity change characteristics (here, the introduction of coefficient γ), so the freeze–thaw damage factor $W_{t}$ can be expressed as

(18) $$W=\gamma W_{t}$$

(19) $$\gamma =1-\frac{D_{0,min}}{D_{N,min}}$$

In order to investigate the relationship between the peak intensity correlation coefficient and the freeze–thaw damage factor, the model studied by Gao, F et al. [36] was fitted.

(20) $$\frac{\sigma_{N}}{\sigma_{0}}=\eta \exp\left(-\rho \Delta W\right)$$

where η and ρ are the correlation coefficients and ∆W is the change in the freeze–thaw damage factor $∆W=W_{N}-W_{0}$.

**Figure 13:**
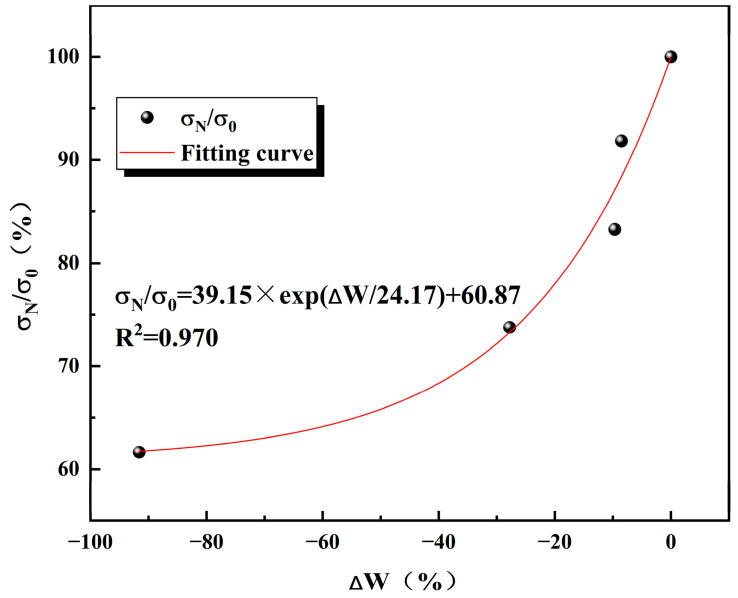
Peak intensity correlation coefficients versus the amount of freeze–thaw variation.

As can be seen from the Figure 13, the two show a pattern of exponential function growth, and $R^{2}$ equals 0.970. This is in high agreement with the model [36] and η equals 39.15 and ρ equals −1/24.17. Therefore, the use of porosity to evaluate freeze–thaw cycling damage in sandstone is feasible and applicable to other rocks.

#### 3.3.2. Scanning Electron Microscope

**Figure 14:**
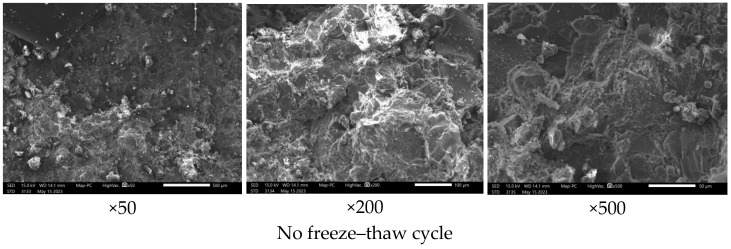

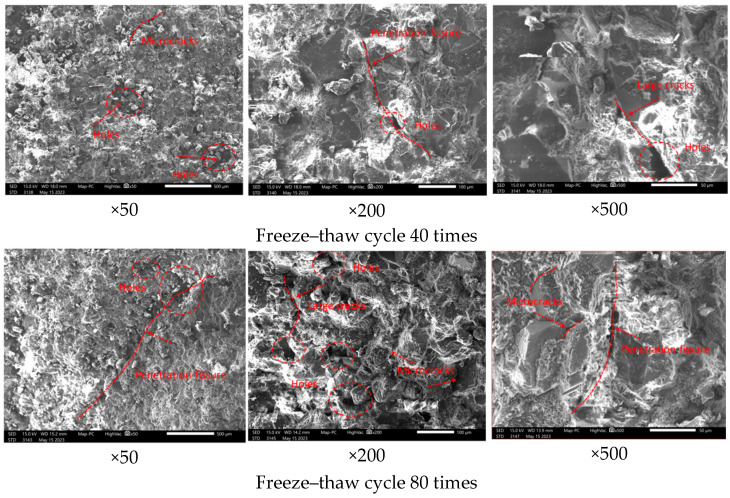
SEM under different numbers of freeze–thaw cycles.

Sandstones are composed of quartz, sodium feldspar, calcite, hematite and other minor components, and they generally have a porosity of about 10~25% [37]. Fragments of sandstone from the unfrozen–thawed, 40 cycles of freeze–thawing and 80 cycles of freeze–thawing were taken, and the internal microstructure of the sandstone was observed at magnifications of 50×, 200× and 500×; see Figure 14. There is a more pronounced change in freeze–thaw damage. It was observed that the sandstone without freeze–thawing was dense and intact, with few holes and micro-fractures visible. At 50× magnification, a few micro-pores and micro-fissures are seen in the sandstone with 40 freeze–thaw cycles; at 200× magnification, obvious through-fissures and pores are observed, and at 500× magnification, larger pores and large fissures are found. At 50× magnification, the sandstone with 80 freeze–thaw cycles has obvious pores and through-fissures compared to the sandstone with no freeze–thaw and 40 freeze–thaw cycles; at 200× magnification, as the number of freeze–thaw cycles increases, more localized pores and micro-fissures appear, with a loose structure forming a honeycomb; at 500× magnification, obvious through-fissures and a few micro-fissures are observed, with a large number of micro-fissures gradually developing to form a large number of micro-fractures that gradually develop into large fissures and extend, expand and penetrate to the periphery. The reason for this is that the water–ice phase change and repeated freezing and melting have weakened the cementation between the sandstone grains and damaged the internal microstructure and integrity of the sandstone, and the damage is mainly in the form of cross-grain, along-grain and tangential fractures.

### 3.4. Damage Evolution

#### 3.4.1. Damage Model under the Fatigue Loading of Rock

The Weibull probability density function is introduced by statistically modeling the damage based on various defects within the rock.

(21) $$P\left(\varepsilon \right)=\frac{m}{F}{\left(\frac{\varepsilon }{F}\right)}^{m-1}exp\left[-{\left(\frac{\varepsilon }{F}\right)}^{m}\right]$$

In the form, $P\left(\varepsilon \right)$ and ε are the internal probability distribution and microstrain of the rock under micro-stress, respectively, and m and F are the distribution parameters.

The micro-cracks within the rock under external loading $N_{s}$ occur in large numbers.

(22) $$N_{s}=\int_{0}^{\varepsilon }NP\left(x\right)dx=N\left\{1-exp\left[-{\left(\frac{\varepsilon }{F}\right)}^{m}\right]\right\}$$

Damage to the rock is the cause of an increasing number of micro-cracks in the interior, as a consequence of defining a damage model that varies between 0 and 1.

(23) $$D=\frac{N_{s}}{N}$$

The damage evolution equation is obtained by substituting Equation (22) into Equation (23).

(24) $$D=1-exp\left[-{\left(\frac{\varepsilon }{F}\right)}^{m}\right]$$

The theory of loading and unloading response ratios proposed by Yin et al. [38] can be used to study the precursors of instability in nonlinear systems with the following equation.

(25) $$Y_{E}=\frac{E^{+}}{E^{-}}$$

where *E* and *F* are the modulus of elasticity in the loading phase and the modulus of elasticity in the unloading phase, respectively.

According to Shi et al. [39], Equation (25) can be further optimized into the following equation.

(26) $$Y_{E}=\frac{E^{+}}{E^{-}}=\frac{1}{1-\frac{\varepsilon }{\left(1-D\right)}\frac{dD}{d\varepsilon }}$$

The derivation of Equation (24) yields

(27) $$\frac{dD}{d\varepsilon }=\left(D-1\right)\left[-\frac{m}{F}{\left(\frac{\varepsilon }{F}\right)}^{m-1}\right]$$

The damage within the rock accumulates as it is loaded and unloaded, so the damage variables are also cumulative, and substituting Equation (27) into Equation (26) yields

(28) $$D=\sum_{i=1}^{n}D_{i}=\sum_{i=1}^{n}\left(1-e^{\frac{Y_{E(i)}-1}{mY_{E(i)}}}\right)$$

Based on the basic principles of damage mechanics, the rock damage model equations are as follows:

(29) $$\sigma =E\varepsilon \left(1-D\right)$$

Substituting Equation (21) into Equation (25) and deriving it yields

(30) $$\frac{d\sigma }{d\varepsilon }=Ee^{-\left(\frac{\varepsilon }{F}\right)m}\left[1-\frac{\varepsilon m}{F}{\left(\frac{\varepsilon }{F}\right)}^{m-1}\right]$$

when $\sigma =\sigma_{p}$, $\varepsilon =\varepsilon_{p}$; therefore, $\frac{d\sigma }{d\varepsilon }=0$.

$\sigma_{p}$ and $\varepsilon_{p}$ are the peak strength and peak strain, respectively.

Accordingly, the parameter *F* is obtained.

(31) $$F=\frac{\varepsilon_{p}}{{\frac{1}{m}}^{\frac{1}{m}}}$$

Substituting $\sigma =\sigma_{p}$ and $\varepsilon =\varepsilon_{p}$ into Equation (29) yields

(32) $$\sigma_{p}=E\varepsilon_{p}\left(1-D\right)=E\varepsilon_{p}e^{-\frac{1}{m}}$$

Combining Equations (31) and (32) yields the following equation:

(33) $$m=-\frac{1}{\ln\frac{\sigma_{p}}{E\varepsilon_{p}}}$$

(34) $$F=\frac{\varepsilon_{p}}{{\left(-\ln\frac{\sigma_{p}}{E\varepsilon_{p}}\right)}^{-\ln\frac{\sigma_{p}}{E\varepsilon_{p}}}}$$

#### 3.4.2. Damage Model under the Coupled Freeze–Thaw of Rock

The internal structure of the rock is damaged by the freezing and swelling forces, weakening the rock’s ability to withstand them. Because of the complexity of the microscopic mechanisms of freeze–thaw damage, the extent of freeze–thaw damage is currently only reflected from a macroscopic perspective. The modulus of elasticity of rock is a good indicator of the deformation of rock and its ability to resist external forces, so it is usually used as a criterion for judgement.

(35) $$D_{n}=1-\frac{E_{n}}{E_{0}}$$

where $D_{n}$ is the freeze–thaw damage factor and $E_{0}$ and $E_{n}$ are the moduli of elasticity of the rock before and after freeze–thawing, respectively.

#### 3.4.3. Damage Model under the Coupled Freeze–Thaw-Fatigue Loading of Rocks

According to the Lemaitre strain equivalence principle, freeze–thaw damage to rock is regarded as the first damage state, and freeze–thaw damage and fatigue load damage to rock are regarded as the second damage state. Then, the two damage constitutive equations are as follows:

(36) $$\sigma_{n}=\left(1-D_{n}\right)E\varepsilon_{n}$$

(37) $$\sigma =\left(1-D\right)E_{n}\varepsilon$$

The principal structure relationship under coupled freeze–thaw-fatigue loading is obtained by combining Equation (36) and Equation (37).

(38) $$\sigma =\left(1-D\right)\left(1-D_{n}\right)E_{0}\varepsilon$$

The damage model under coupled freeze–thaw-fatigue loading is obtained from Equation (31).

(39) $$D_{t}=D+D_{n}-DD_{n}$$

where $D_{t}$ is the damage factor under coupled freeze-thaw-fatigue loading; D is the damage factor under fatigue loading; $D_{n}$ is the freeze-thaw damage factor.

## 4. Conclusions

The mechanical properties of sandstone under coupled freeze–thaw-fatigue action were analyzed and a damage model was developed. The studies of the microstructural changes in freeze–thaw damage led to the following main conclusions:

(1) The higher the number of freeze–thaw cycles, the lower the peak strength, frost resistance, modulus of elasticity, modulus of deformation and damping ratio; as the load cycle level increases, the modulus of deformation and modulus of elasticity increase non-linearly, the rate of increase gradually decreases, the dissipation energy due to hysteresis gradually increases and the rate of increase becomes faster and faster, while the overall damping ratio shows a pattern of gradually decreasing and increasing at a later stage.
(2) As the number of freeze–thaw cycles increases, the total porosity and micro-porosity of the sandstone increase almost linearly, and the micro-porosity is more sensitive to the effects of freeze–thaw, shifting to medium and large pores, and it is found by SEM that the higher the number of freeze–thaw cycles of the sandstone, the more micro-fractures and pores develop and the more loose the structure is, and the whole is in the shape of a nesting bee.
(3) Based on the sandstone pore fractal theory, it is found that $D_{min}$ is more sensitive to freeze–thaw; thus, the study of freeze–thaw damage evolution law needs to consider the micro-pore distribution characteristics as well as the complexity, and based on the loading and unloading response ratio theory and strain equivalence principle, a damage model under coupled freeze–thaw-fatigue loading was established.

## Figures and Tables

**Table 1:** Basic Mechanical Parameters of Sandstone.

Compressive Strength (MPa)	Modulus of Elasticity (GPa)	Tensile Strength (MPa)	Poisson’s Ratio ν
44.72	3.72	2.57	0.30

**Table 2:** Peak strength and breakage level of specimens.

Number of Freeze–Thaw Cycles	Specimen Number	$\mathrm{Peak}\;\mathrm{Strength}\;\sigma_{p}$ (MPa)	$\mathrm{Average}\;\mathrm{Peak}\;\mathrm{Strength}\;\sigma_{p}\;$ (MPa)	Breakage Level
0	1	48.98	49.95	12
	2	49.20		
	3	51.59		
20	1	44.99	45.48	11
	2	45.25		
	3	46.2		
40	1	41.40	41.43	10
	2	40.99		
	3	41.9		
60	1	36.2	36.4	9
	2	36.7		
	3	36.3		
80	1	30.88	31.17	7
	2	31.7		
	3	30.93		

**Table 3:** NMR fractal dimension.

Fractal Dimension	Number of Freeze–Thaw Cycles				
	0	20	40	60	80
$D_{min}$	0.629	0.547	0.629	0.547	0.628
$D_{max}$	2.981	2.978	2.981	2.978	2.981

## Data Availability

Data are contained within the article.
